# The forkhead box containing transcription factor FoxB is a potential component of dorsal-ventral body axis formation in the spider *Parasteatoda tepidariorum*

**DOI:** 10.1007/s00427-020-00650-z

**Published:** 2020-02-07

**Authors:** Miriam Heingård, Ralf Janssen

**Affiliations:** 1 Department of Earth Sciences, Palaeobiology, Uppsala University, Villavägen 16, Uppsala, Sweden; 2 Department of Geology, Faculty of Science, Lund University, Sölvegatan 12, Lund, Sweden

**Keywords:** Arthropoda, Axis formation, Development, Dorsal, Ventral, Dorsoventral patterning, germ band formation

## Abstract

In the spider, determination of the dorsal-ventral body (DV) axis depends on the interplay of the dorsal morphogen encoding gene *decapentaplegic* (*Dpp*) and its antagonist, *short gastrulation* (*sog*), a gene that is involved in the correct establishment of ventral tissues. Recent work demonstrated that the forkhead domain encoding gene *FoxB* is involved in dorsal-ventral axis formation in spider limbs. Here, Dpp likely acts as a dorsal morphogen, and FoxB is likely in control of ventral tissues as RNAi-mediated knockdown of *FoxB* causes dorsalization of the limbs. In this study, we present phenotypes of *FoxB* knockdown that demonstrate a function in the establishment of the DV body axis. Knockdown of FoxB function leads to embryos with partially duplicated median germ bands (*Duplicitas media*) that are possibly the result of ectopic activation of Dpp signalling. Another class of phenotypes is characterized by unnaturally slim (dorsal-ventrally compressed) germ bands in which ventral tissue is either not formed, or is specified incorrectly, likely a result of Dpp over-activity. These results suggest that FoxB functions as an antagonist of Dpp signalling during body axis patterning, similarly as it is the case in limb development. FoxB thus represents a general player in the establishment of dorsal-ventral structures during spider ontogeny.

**Electronic supplementary material:**

The online version of this article (10.1007/s00427-020-00650-z) contains supplementary material, which is available to authorized users.

## Introduction

**Fig. 1:**
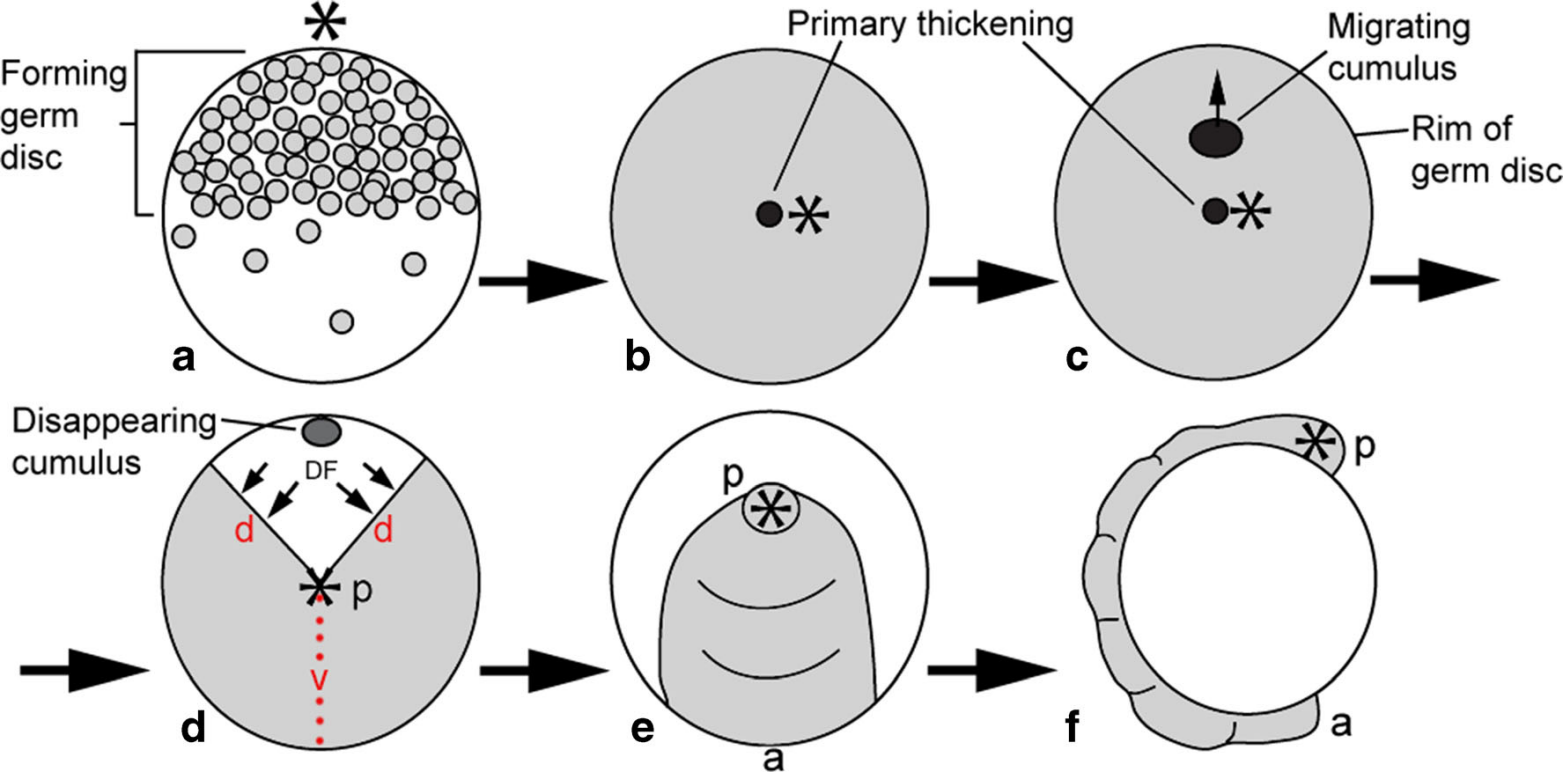
Formation of the spider germ band. In all panels, the asterisks mark the posterior pole of the developing embryo, the centre of the germ disc. **A** Lateral view. Formation of the germ disc as cells accumulated in one half of the egg. **B** On-top view on the germ disc. Formation of the primary thickening. **C** On-top view on the germ disc. The cumulus moves to the periphery of the germ disc. **D** On-top view on the germ disc. The dorsal field (DF) is induced as the cumulus reaches the rim of the disc. Arrows mark direction of DF expansion. The dotted line marks the future ventral midline of the forming germ band. **E** On-top view on the germ disc. The germ band has formed. The rim of the disc represents “anterior”. **F** Lateral view. Elongation of the germ band and formation of segmental grooves. Abbreviations: *a* anterior, *d* dorsal, *p* posterior, *v* ventral

The development of spiders is the subject of intensive investigation on both morphological and molecular level, with *Parasteatoda tepidariorum* (earlier syn. *Achaearanea tepidariorum*) representing the main model species (e.g. Montgomery Jr 1909; Holm 1940, 1952; Sekiguchi 1957; Seitz 1966; Suzuki and Kondo 1995; Stollewerk et al. 2001; Damen 2002; Mittmann and Wolff 2012; Khadjeh et al. 2012; Kanayama et al. 2010; Schwager et al. 2015, 2017; Pechmann et al. 2017; Leite et al. 2018; Oda et al. 2019). A crucial step in spider development is the formation of a radial disc of cells on top of the yolk, the germ disc (e.g. Holm 1952; Akiyama-Oda and Oda 2003; Wolff and Hilbrant 2011; Mittmann and Wolff 2012; Oda et al. 2019). In the centre of this disc forms the so-called cumulus, a “primary thickening” of mesenchymal cells (e.g. Holm 1952; Akiyama-Oda and Oda 2003). From here, a portion of these cells move to the periphery of the disc. Where the cells reach the rim of the disc, the disc “opens” and cells move circumferentially towards the opposite side of the disc. In following stages, the embryo equals a closing fan, whereby the outer rim of the fan will become the anterior pole of the embryo, and the centre of the disc, the hinge of the fan, will become the posterior pole of the embryo. The lateral sides of the fan become dorsal, and the central tissue between the hinge and the periphery become the ventral midline (e.g. Holm 1952, Akiyama-Oda and Oda 2003) (Fig. 1).

Recent studies in *Parasteatoda* revealed that the moving cumulus expresses inter alia the dorsal morphogen encoding gene *decapentaplegic* (*dpp*), and knockdown of Dpp function caused the development of embryos with persisting radial symmetry strongly implying that Dpp functions as a key-factor in the transformation from radial to bilateral symmetry in spider development. Activity of Dpp induces also the formation of the so-called dorsal field. The interface between the germ band proper and the dorsal field represents the most dorsal of the developing embryo; Dpp thus acts as a dorsal morphogen (Akiyama-Oda and Oda 2003, 2006). The available morphological and developmental data describing the processes that govern the transition from the radial germ disc of spiders into the bilateral germ band are reviewed and further investigated in two papers published in this special issue of Development Genes and Evolution (Oda et al. 2019; Pechmann (2020).

Overall, these findings are in line with the function of Dpp in *Drosophila* and other animals such as vertebrates (the vertebrate ortholog of Dpp is Bmp2/4) where these genes act as dorsal or ventral morphogens in the establishment of the primary body axis and the development of the limbs (e.g., Irish and Gelbart 1987; St Johnston and Gelbart 1987; Holley et al. 1995; Arendt and Nübler-Jung 1997).

In a recent paper, we described the forkhead domain transcription factor FoxB as a key regulator of dorsal-ventral limb patterning in *Parasteatoda* (Heingård et al. 2019). Knockdown of FoxB function via parental RNA interference (RNAi) causes the limbs to lose their ventral identity: genes expressed in the ventral ectoderm of the limbs disappeared from *FoxB* knockdown embryos, while genes expressed along the dorsal side of the limbs invaded ventral territories (Heingård et al. 2019). *Dpp*, which is expressed in the tips of the developing appendages in wild type embryos, expanded its expression into the ventral limb ectoderm. These data strongly suggest that FoxB plays a role in orchestrating dorsal vs ventral cell identity in the limbs. FoxB likely controls activity of ventral genes such as *wingless* (*wg*) and *H15* and represses expression of dorsal genes such as *optomotor-blind* (*omb*) and *Dpp* (Heingård et al. 2019).

The knockdown of *FoxB*, however, also causes germ band-specific phenotypes in the form of a partially duplicated germ band (the Class-III phenotype: *Duplicitas media*) and embryos with an unnaturally slim germ band (Class-II phenotype). Both such phenotypes are congruent with a conserved function of FoxB as a repressor of Dpp signalling and thus dorsal-ventral (DV) patterning during early germ band formation. FoxB thus appears to be responsible for the establishment of DV symmetry in appendages (Heingård et al. 2019) as well as in the main body axis.

## Methods

### Animal husbandry and developmental staging

*Parasteatoda* spiders were obtained from the colony in Göttingen, Germany. The spiders were kept separately in plastic vials at room temperature (approximately 20–21 °C), supplied with water daily and fed with either subadult *Acheta domesticus* or *Drosophila melanogaster* flies. Developmental staging is after Mittmann and Wolff (2012).

### Phylogenetic analysis, gene cloning, whole mount in situ hybridization, nuclear staining and parental RNAi

Phylogenetic analysis of panarthropod FoxB genes is published, and cloning of *Parasteatoda FoxB* is explained in Heingård et al. (2019). A fragment of *FoxB* was isolated by means of RT-PCR with gene-specific primers based on sequence information from the sequenced genome (Schwager et al. 2017). We applied the whole mount in situ hybridization protocol as desribed in Janssen et al. (2018). Cell nuclei were visualized incubating embryos in 3 μg/ml of the fluorescent dye 4-6-Diamidino-2-phenylindole (DAPI) in phosphate-buffered saline with 0.1% Tween 20 (PBST) for approximately 20–30 min. Excess of DAPI was washed away with PBST.

Double-stranded RNA (dsRNA) (in vitro transcribed as described in Heingård et al. 2019) was injected laterally into the opisthosoma of adult females. We performed two independent rounds of injection each with freshly prepared dsRNA. Each spider was injected three times (on three consecutive days) with each time 2.5 μl of 2.8 μg/μl dsRNA or 4 μg/μl dsRNA in injection buffer (1.4 mM NaCl, 0.07 mM Na_2_HPO_4_, 0.03 mM KH_2_PO_4_, 4 mM KCl). The concentration of injected *FoxB* dsRNA did not significantly influence the outcome of the experiments. Control spiders were injected with 2.5 μl of injection buffer. We injected 20 adult females with *Pt-FoxB* dsRNA in injection buffer and 20 adult females with only injection buffer. Cocoons of dsRNA-injected spiders were investigated separately, and all available control cocoons were pulled and investigated as one batch. Embryos with different morphologically distinguishable phenotypes were categorized into distinct classes of which two, Class-II and Class-III, are of particular interest for this study. The Class-I knockdown phenotype has been described in Heingård et al. (2019).

## Results

### Knockdown of *FoxB*

The data presented in this paper are based on the experiments conducted and described in Heingård et al. (2019).

**Fig. 2:**
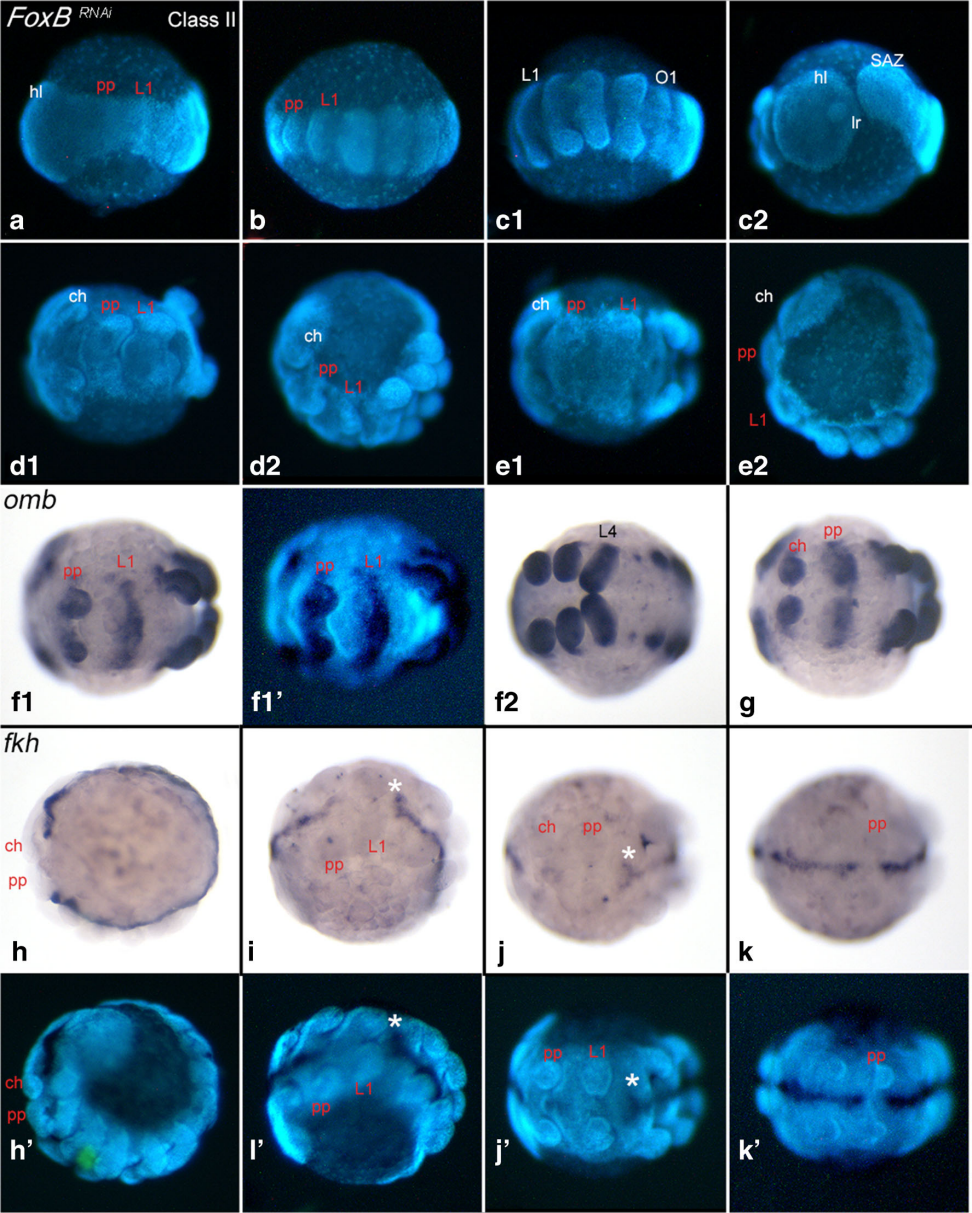
Class-II embryos – unnaturally slim germ bands. Same letters represent same embryo. Letter with different roman numerals represent different views on the same embryo. In all panels, anterior is to the left, ventral views. Panels **A**–**E2**, **F1´** and **H´**–**K´** show DAPI-stained embryos. Panel letters marked with an apostrophe (´) represent DAPI-stained embryos as seen in corresponding bright field images (without apostrophe). Embryos in panels **F1**, **F2** and **G** are stained for *optomotor-blind* (*omb*). Embryos in panels **H**–**K** are stained for *forkhead* (*fkh*). White asterisks (*) in panels **I´** and **J´** mark branching expression at the interface of *fkh*-expressing and *fkh*-negative tissue along the ventral midline. Red segment-abbreviations indicate segments in which expression of the ventral midline marker is missing. Abbreviations: *ch* chelicera, *hl* head lobe, *L* walking-leg, *lr* labrum, *pp* pedipalp, *SAZ* segment addition zone

**Fig. 3:**
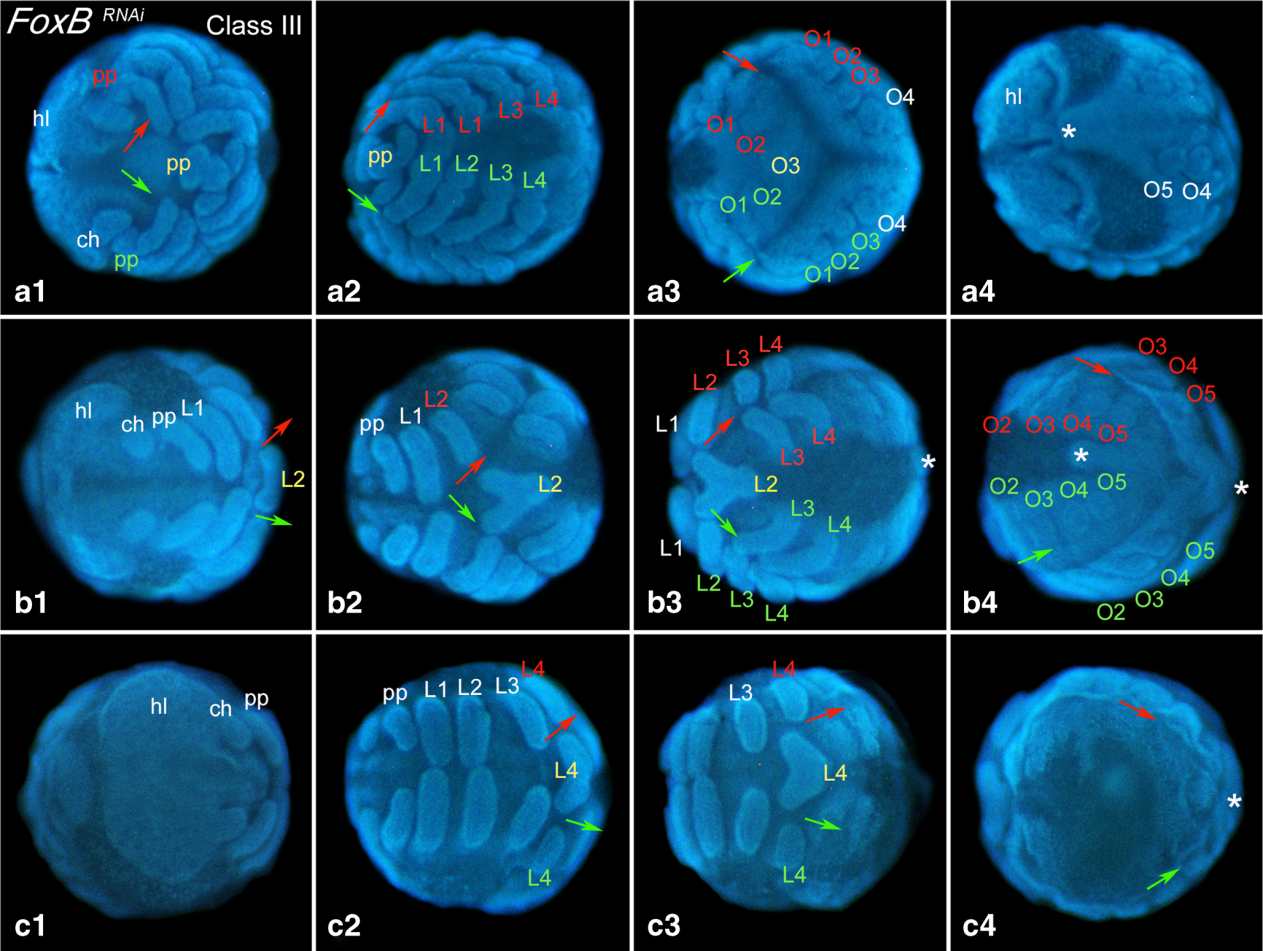
Class-III embryos – duplicated median germ bands (*Duplicitas media*) All embryos are DAPI stained. Same letter indicates same embryo; roman numerals in combination with letter indicated different views on the same embryo. White asterisks (*) mark the posterior end of embryos. Red and green arrows (and associated red and green labels) mark the directions of the duplicated median germ bands. Yellow labels indicate fused (shared) structures of duplicated germ bands. Abbreviations as in Fig. 2; *O* opisthosomal segment

Hatching rates are significantly decreased in *FoxB* knockdown embryos suggesting severe effects of *FoxB* downregulation on development. Altogether, we described four different classes of phenotypes. Class-I embryos possess abnormally developed limbs (described in Heingård et al. 2019). Class-II embryos are characterized by an unnaturally slim germ band (Fig. 2). Class-III embryos, which indeed are very rare compared to the other observed phenotypes (46/3680 embryos (=1.25%)) (Supplementary Fig. S1), develop a medially duplicated germ band (*Duplicitas media*) (Fig. 3). Although the number of embryos with the Class-III phenotype are rare, we observed this phenotype in cocoons from different *FoxB*-injected female spiders and in both of the experimental setups we conducted. A shortcoming of our study is the lack of a control conducted with an independent fragment of the *FoxB* transcript. This offers the possibility of so-called off-target effects. In theory, the Class-III phenotype could be caused by such an off-target effect and thus be correlated with the function of another gene. The occurrence of partially duplicated germ bands after the knockdown of *FoxB*, however, is in line with the suggested function of FoxB as a repressor of Dpp or Dpp signalling (Heingård et al. (2019), and discussed below). Class-IV embryos do not develop beyond the formation of an (often heavily malformed) germ disc (not shown). Approximately 10% of all embryos represent either unfertilized eggs or embryos that died shortly after fertilization. In either case, such embryos never developed a protective vitelline membrane and therefore appeared as hardened yolk masses after fixation. Numbers of dead embryos equal those in wild type cocoons.

In our controls, we recognized a small percentage of Class-II and Class-IV embryos indicating that formation of the germ disc and transition from radial to bilateral symmetry represent critical steps in spider development (Heingård et al. 2019). However, we did not find a single Class-III embryo in our control embryos. Also, as far as we know, there is no case in the scientific literature describing a naturally occurring similar abnormally formed germ band as seen in Class-III embryos. Occasionally, spider embryos develop two anterior poles (*Duplicitas anterior*) (Oda and Akiyama-Oda 2008), or after RNAi treatment of genes involved in the formation and maintenance of the posterior segment addition zone (SAZ), can develop two (or more) posterior poles (*Duplicitas posterior*) (McGregor et al. 2008).

The germ band of Class-II embryos is slim when compared with embryos of comparable developmental stage. Reduction of the dorsal-ventral extension of the germ band includes the complete embryo. However, the chelicera-, pedipalp-, and first leg-bearing segments appear to be more affected than the head lobes and more posteriorly located segments (Fig. 2). In Class-II embryos, *optomotor-blind* (*omb*) is expressed in transverse stripes in these segments (Fig. 2f/g), instead of in the form of two ventrally separated domains as it is the case in wild type embryos (e.g., Akiyama-Oda and Oda 2006). In these most dorsal-ventrally condensed segments, also the ventral midline marker *forkhead* (*fkh*) is not expressed, while expression is normal in the head lobes and the other segments (Fig. 2h–k). Occasionally, expression of *fkh* is in the form of a Y-shaped expression at the interface between midline tissue that expresses the marker and “midline” tissue that does not express the marker (Fig. 2i/j).

**Fig. 4:**
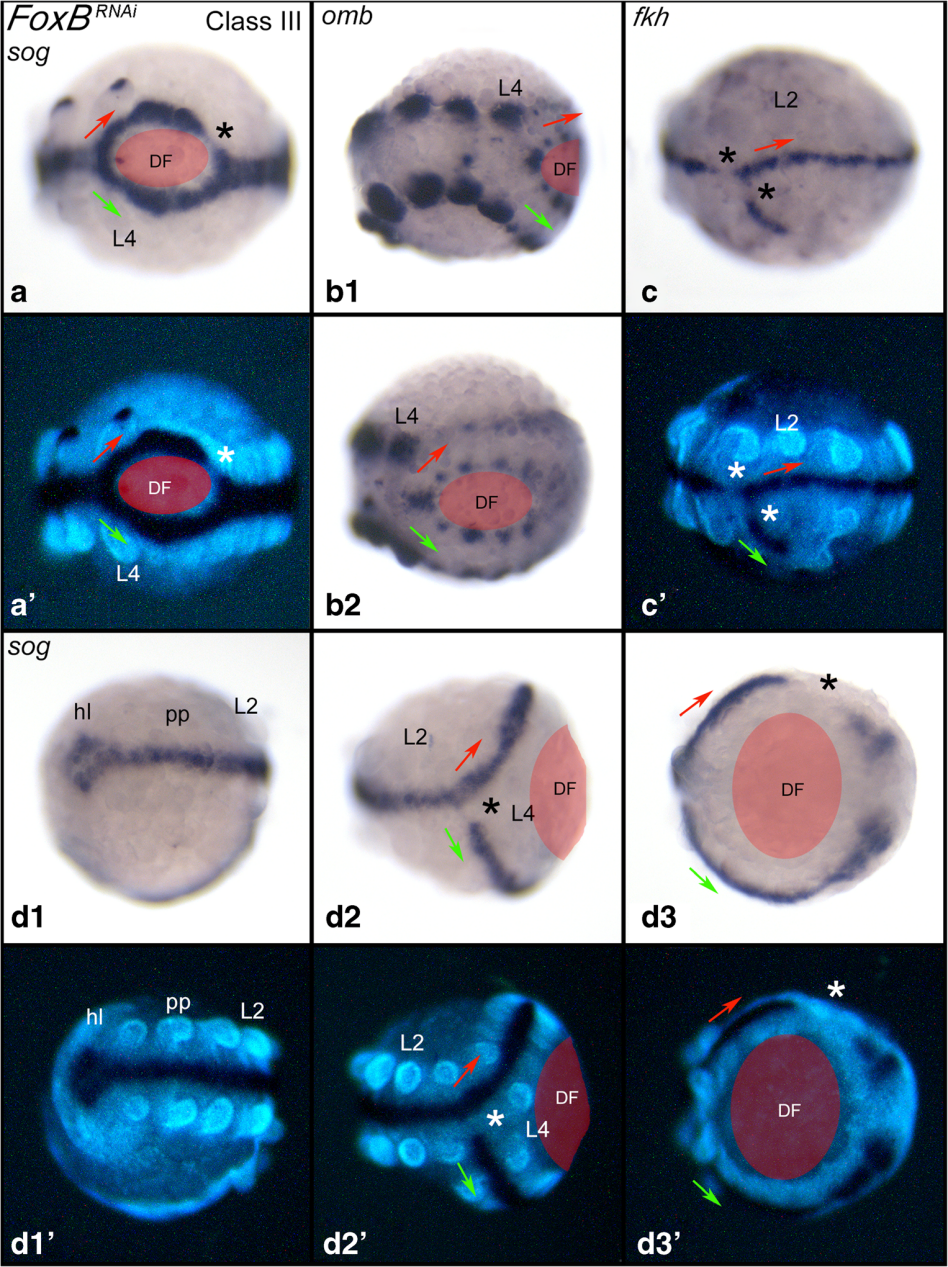
Marker-gene expression in Class-III embryos. In all panels, anterior is to the left, ventral views. Red and green arrows mark direction of duplicated germ bands. Ectopic dorsal fields (DF) are indicated by red shades. In all panels, asterisks mark lacking marker-gene expression in the ventral midline. Embryos shown in panels **A**, and **D1**–**D3** are stained for *short gastrulation* (*sog*); embryo shown in panels **B1**/**B2** are stained for *optomotor-blind* (*omb*); embryo shown in panel **C** is stained for *forkhead* (*fkh*). Abbreviations as in Fig. 2

The germ band of Class-III embryos is partially duplicated. However, the duplicated tissue never includes either two separate heads (*Duplicitas anterior*) or two separate tails (*Duplicitas posterior*). Instead, at least the most anterior and the most posterior region of the embryo are shared by the two medially duplicated germ bands (Fig. 3). Duplication of the anterior-posterior body axis (AP axis) appears in variable position in Class-III embryos. Examples shown in Fig. 3 represent one embryo with duplications reaching from the pedipalp-bearing segment to the third opisthosomal segment (Fig. 3a), one embryo with duplicated tissue from the second walking-leg-bearing segment to the posterior SAZ (Fig. 3b) and one embryo with duplication from the fourth walking-leg-bearing segment to the SAZ (Fig. 3c). The embryo with the largest found-duplicated region only shares part of the anterior head lobes; all other segments are duplicated except for the most posterior region of this embryo (not shown). In some Class-III embryos, the germ band is only mildly duplicated. Those embryos are more difficult to recognize as they only comprise a somewhat broadened median body region. We used the midline markers *fkh* and *short gastrulation* (*sog*) and *omb* to visualize duplication events in these embryos (Fig. 4a/b). In one embryo, we found a rudimentary-duplicated region that expressed the midline marker *fkh*, but this rudimentary duplicated region ended blind (and was thus not connected with the posterior of the embryo) (Fig. 4c). Notably, often the region anterior and/or posterior adjacent to the region where the midline splits (and the duplicated area begins or ends) do not express midline markers (Fig. 4a, c, d). In one embryo, we also found disturbed midline expression in one of the duplicated posterior germ bands (Fig. 4d).

## Discussion

### Class-III *FoxB*-knockdown embryos: medially duplicated germ bands (*Duplicitas media*) as the result of the formation of an ectopic dorsal field (DF)

**Fig. 5:**
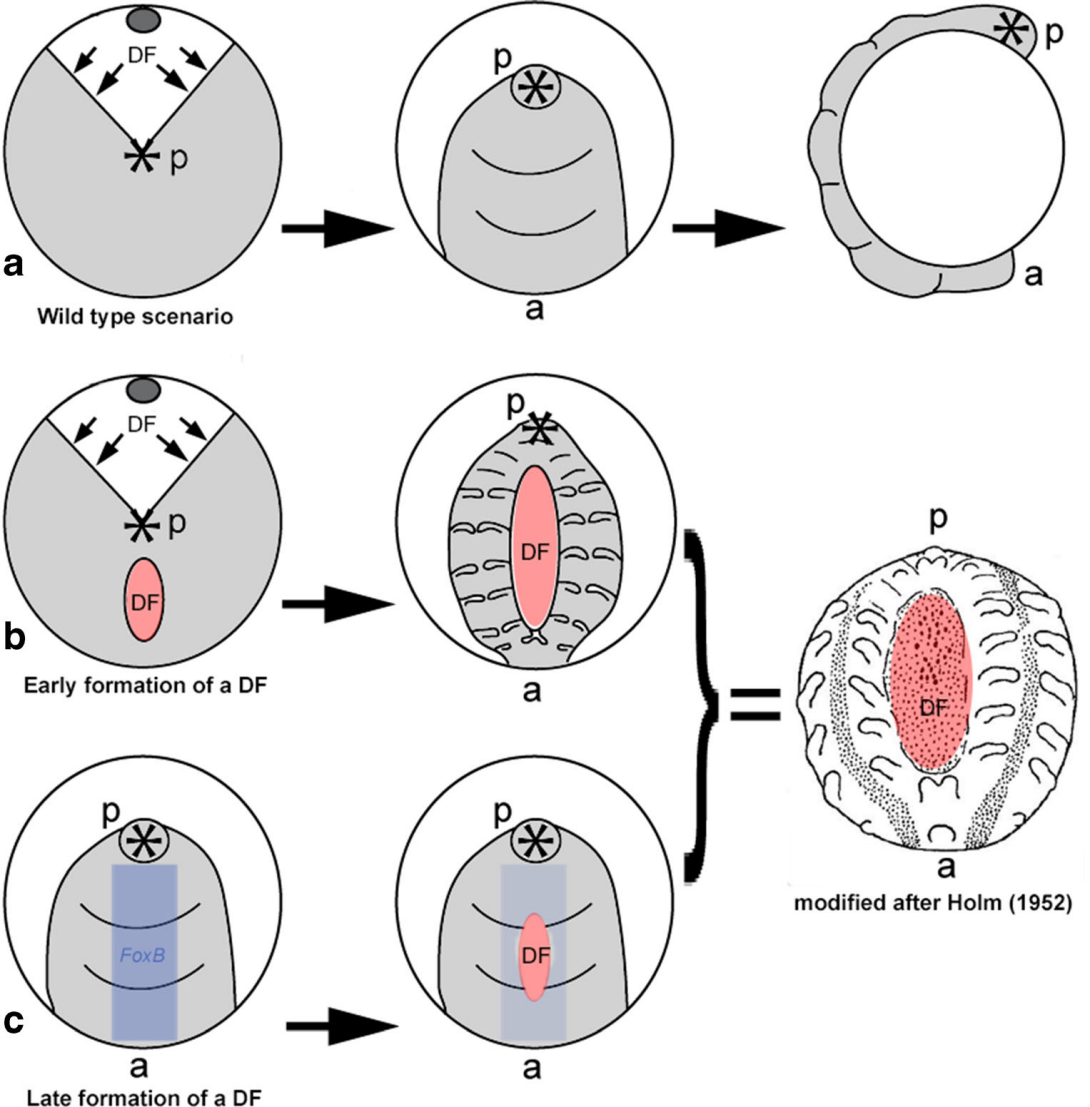
Formation of Class-III embryos. Abbreviations as in Fig. 1. (**A**) Normal development and the initiation of one dorsal field (DF) leading to the formation of a regular germ band. Last panel of this row shows a lateral view (**B**) Ectopic induction of a secondary DF (red shades) at the germ disc stage that leads to the formation of a partially duplicated median germ band. The drawing of the embryo to the right (middle) represents a theoretic scenario, not proven by the provided data (**C**) Ectopic induction of a secondary DF (red shades) at the early germ band stage. Wild type expression of *FoxB* is indicated (deep blue) in the first panel of this row; in the second panel, *FoxB*-knockdown is indicated (light blue). The drawing of the embryo to the right (middle) represents a theoretic scenario, not proven by the provided data. The last panel of the figure is modified after Holm (1952) showing the partially duplicated median germ band.

Transformation of the circular germ disc into a bilaterally symmetric germ band is controlled by mesenchymal cumulus cells that originate from the centre of the disc (the future posterior pole of the embryo) and move towards the rim of the disc. On their way, these cells express *dpp*, and this causes the germ disc to “open” (Figs. 1 and 5a). At the same time, Dpp induces the development of dorsal cell fate and thus the dorsal field (DF) (Akiyama-Oda and Oda 2003, 2006). When the movement of the cumulus from the centre of the disc to its periphery is inhibited or delayed, ectopic fields form in close proximity around the centre of the cumulus (Akiyama-Oda and Oda 2010). This demonstrates that induction of ectopic DFs is caused by Dpp signalling (as the arrested cumulus cells express *dpp*). The observation of Class-III embryos after *FoxB* knockdown is thus well-explained by ectopic activity of *dpp* in these embryos. Previous work has shown that FoxB likely acts as a repressor of *dpp* in the developing spider appendages (Heingård et al. 2019). If this is a general function of FoxB, then knockdown of *FoxB* could activate (or derepress) expression of *dpp* in the germ disc as well. Consequently, ectopic activity of Dpp in the germ disc would lead to the induction of dorsal tissue (an ectopic dorsal field (DF)) and splitting of the germ band wherever Dpp is active (Fig. 5b, the drawing of the embryo to the left represents a theoretic scenario, not proven by the provided data). Indeed, already the work of Åke Holm (1952) showed that transplantation of (*dpp* expressing) cumulus cells leads to the induction of a second AP body axis (reviewed in Oda et al. 2019). In cases where this induction is regionally restricted, as in the place of transplantation in Holm’s grafting experiments, this leads to the formation of an embryo with two medially duplicated germ bands, while the anterior and the posterior regions of the embryo are shared by both partially duplicated germ bands (Holm 1952) (Fig. 5b).

In our experiments, we never observed a case of two separated anterior regions (*Duplicitas anterior*) although this should be the case whenever *dpp* is ectopically activated at the outer rim of the germ band. One explanation for this could be that the periphery of the germ disc is simply not competent for ectopic activation of *dpp* as it is naturally expressed in this tissue (Akiyama-Oda and Oda 2003, 2010). Another reason could be that such scenario did not occur in our experiments, despite being possible; note that the number of Class-III embryos is very low compared to the total number of embryos investigated (Supplementary Fig. S1). The formation of two separate posterior poles, i.e. the splitting of the SAZ, has not been observed.

We also discovered that in all Class-III embryos, the two duplicated median regions are of almost equal quality (except for the embryo shown in Fig. 4c), i.e. they are of the same width and equally well-developed. Likely, this is the result of regulatory events after their formation as spiders are known for their regulative capacity during development (Holm 1952, Sekiguchi 1957, Seitz 1966, 1970, reviewed in Oda et al. 2019).

The germ disc gives rise to all segments of the prosoma (all anterior tissue including the fourth leg-bearing segment (L4)). Opisthosomal segments, however, are generated from the SAZ. Importantly, the most anterior duplicated region of the germ band always lies within the prosoma, concurring with the assumption that formation of an ectopic field is always initiated in the germ disc and that duplication is not induced by any process that occurs during segment addition in the SAZ. However, duplication extends beyond the prosomal segments, which leads to the assumption that the knockdown of *FoxB* and subsequent ectopic activation (de-repression) of *dpp* includes tissue that is generated from the SAZ. Indeed, this is also the case in Holm’s transplantation experiments where duplicated tissue includes opisthosomal segments (Holm 1952, his Fig. 37e). At some point during development, this effect appears to stop and normal (unduplicated) opisthosomal segments are formed (e.g. Figs. 3a and 4a/b) possibly because the effect of the dsRNA-induced *FoxB*-knockdown is wearing off.

Alternatively, induction of an ectopic DF may not occur at the germ band stage, but slightly later, at early stages of germ band formation (Fig. 5c). Compared to the very faint expression of *FoxB* during the germ disc stages, early during germ band formation, around stage 8.1, *FoxB* is strongly expressed along the ventral region of the embryo (Heingård et al. 2019). Again, downregulation of FoxB could cause ectopic activity of Dpp which is otherwise restricted from this area and could thus lead to the formation of a more or less extended duplicated median germ band (Fig. 5c, the drawing of the embryo to the right represents a theoretic scenario, not proven by the provided data). Such later induction of a DF would not require strong regulatory events to form two equally well-developed duplicated median germ bands (as random formation of a DF in the germ disc would likely require). Also in favour of this scenario is the fact that we never observed the formation of a DF in germ disc stage embryos or embryos younger than stage 8.2; however, this could be a statistical artefact due to the low number of embryos showing this phenotype.

### Class-II *FoxB*-knockdown embryos: slim germ bands

In *Drosophila* and spider DV body axis formation, Sog functions as an antagonist of Dpp signalling (Irish and Gelbart 1987; Ferguson and Anderson 1992; François et al. 1994; Akiyama-Oda and Oda 2006; Oda and Akiyama-Oda 2008). In the spider, Dpp also represses the expression of *sog* (Akiyama-Oda and Oda 2006). DV axis formation thus depends on the correct interplay and balance of Sog and Dpp. Interestingly, *Parasteatoda* Class-II *FoxB* knockdown embryos resemble very much those of severe *sog* knockdown embryos (Akiyama-Oda and Oda 2006), implying that both genes, *sog* and *FoxB*, may act in the same gene regulatory network (GRN) that regulates DV axis formation in the spider.

If FoxB, like Sog, inhibits Dpp, then a loss (or reduction) of FoxB activity would increase Dpp signalling, and enhanced Dpp signalling would lead to “dorsalization” of the embryo or the loss (or not formation) of ventral tissue. Indeed, Sog is likely responsible for midline continuity in the spider by repressing Dpp signalling (Oda and Akiyama-Oda 2008), and as our data show, this continuity is disturbed in *FoxB* knockdown embryos and the sog downstream target *fkh* is no longer expressed in the midline (Fig. 2h–k) further supporting this scenario.

### Transition from class-II to class-III phenotypes

Although Class-II and Class-III phenotypes appear to be different, they likely represent different severities of the same genetic disturbance caused by the FoxB knockdown.

Therefore, the Class-II phenotype likely represents a milder form of the Class-III phenotype. The knockdown of FoxB likely leads to overactivity of Dpp signalling (discussed above), and as a result, ventral tissue along the ventral midline does not form or specify properly. If so, the phenotypic expression recognized in Class-II germ bands could easily transform into the Class-III phenotype if ventral tissue is not only disturbed but also acquires dorsal fate. This would possibly lead to the induction of a secondary (ectopic) DF, and the germ band would split. If this is the case, it should be possible to find rare cases of transitional phenotypes in which ventral patterning is disturbed, and induction of an ectopic DF is progressing. We believe that such cases may indeed be represented by the embryos shown in Fig. 2i and j. Especially in the embryo shown in panel j, expression of the ventral marker *fkh* is split at the junction between *fkh*-positive and *fkh*-negative tissue. This may represent the beginning (or the rudiment) of a germ band-splitting process. The number of Class-II phenotype embryos is much larger than that of Class-III embryos (Supplementary Fig. S1). It is thus not unlikely that Class-II embryos represent indeed weaker forms of Class-III embryos.

## Electronic supplementary material

Fig. S1 – Overview over the distribution and numbers of different phenotypes after parental *FoxB* RNAi. Note that Class-I and Class-III phenotypes do not occur in control embryos. The number of Class-II embryos is much enhanced after *FoxB* RNAi (TIF 59670 kb)

High resolution image (PNG 117 kb)

Fig. S2 – Same figure as Fig. 4 but without the overlaying red shades. (TIF 131180 kb)

High resolution image (PNG 1536 kb)
