# Supplementary figures and images for: The forkhead box containing transcription factor FoxB is a potential component of dorsal-ventral body axis formation in the spider Parasteatoda tepidariorum
Source: Dev Genes Evol. 2020 Feb 7;230(2):65–73. doi: 10.1007/s00427-020-00650-z (PMC7128009; doi:10.1007/s00427-020-00650-z)

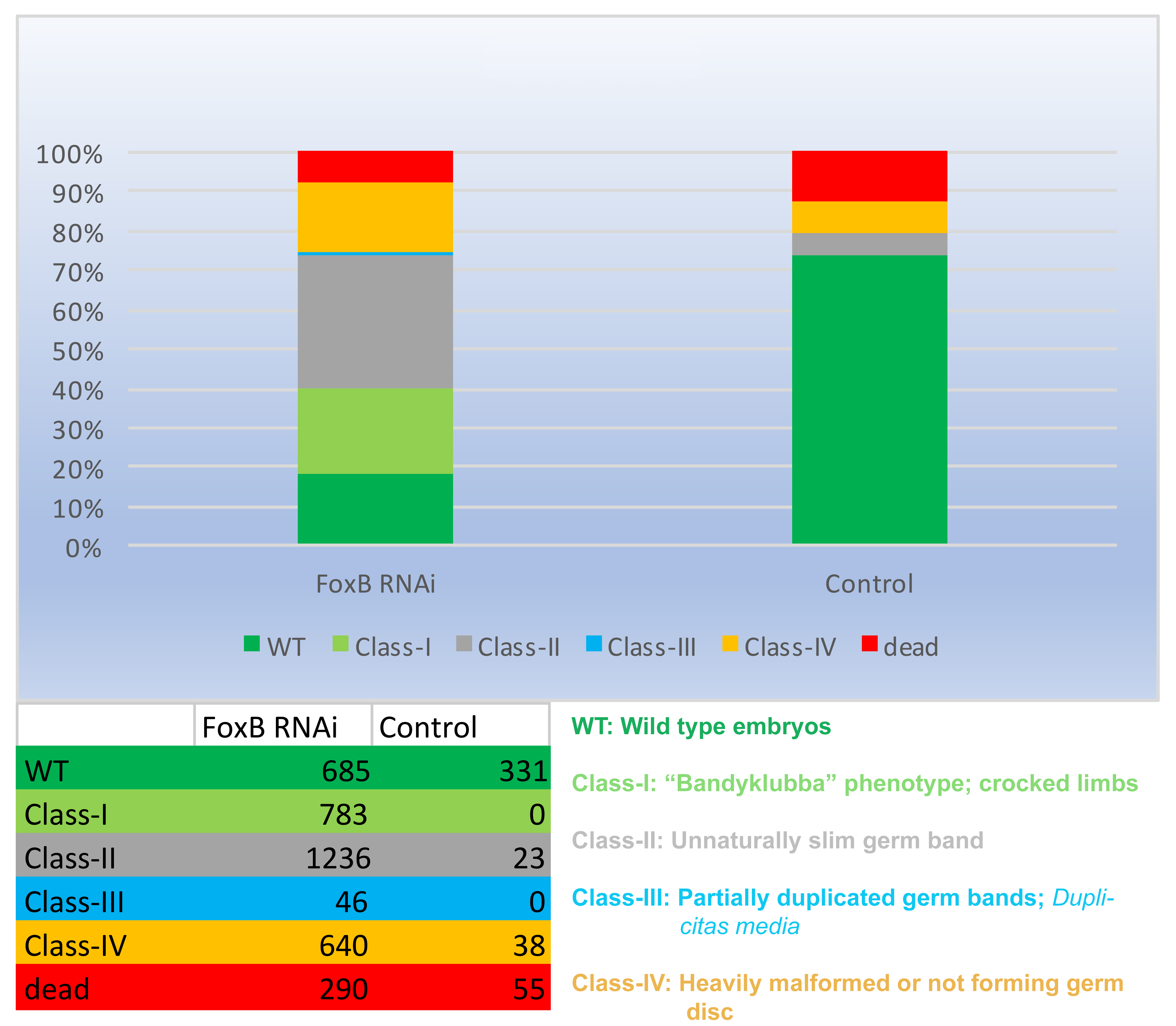

Supplement: Supplementary file 1 — – Overview over the distribution and numbers of different phenotypes after parental FoxB RNAi. Note that Class-I and Class-III phenotypes do not occur in control embryos. The number of Class-II embryos is much enhanced after FoxB RNAi (TIF 59670 kb) [file 427_2020_650_MOESM1_ESM.tif]

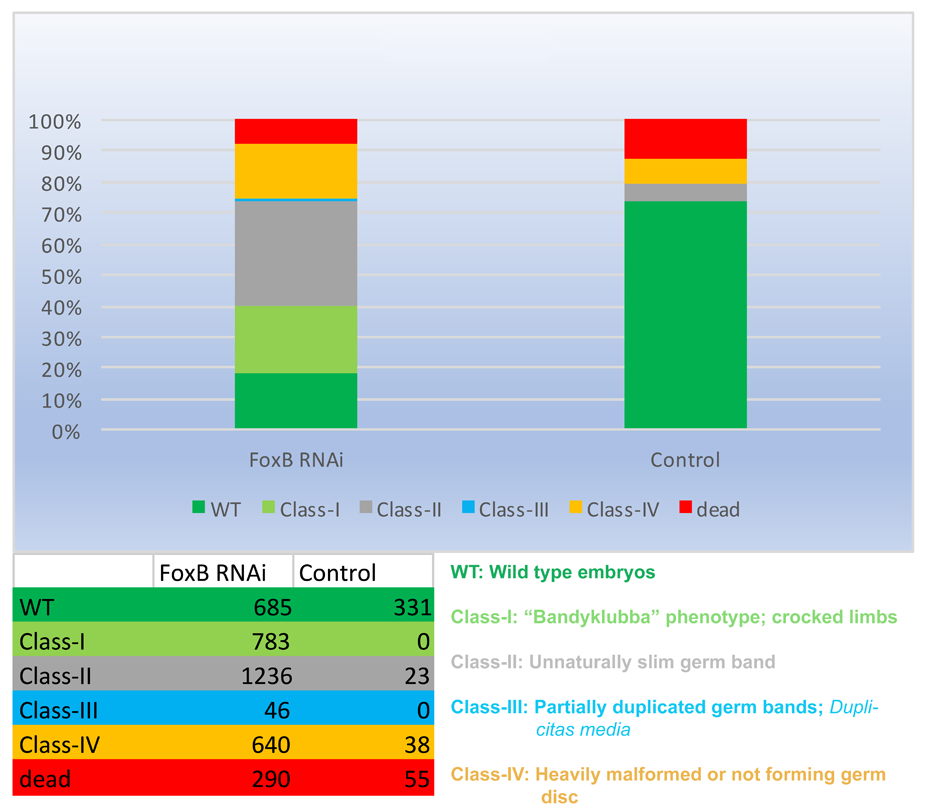

Supplement: Supplementary file 2 — High resolution image (PNG 117 kb) [file 427_2020_650_Fig6_ESM.png]

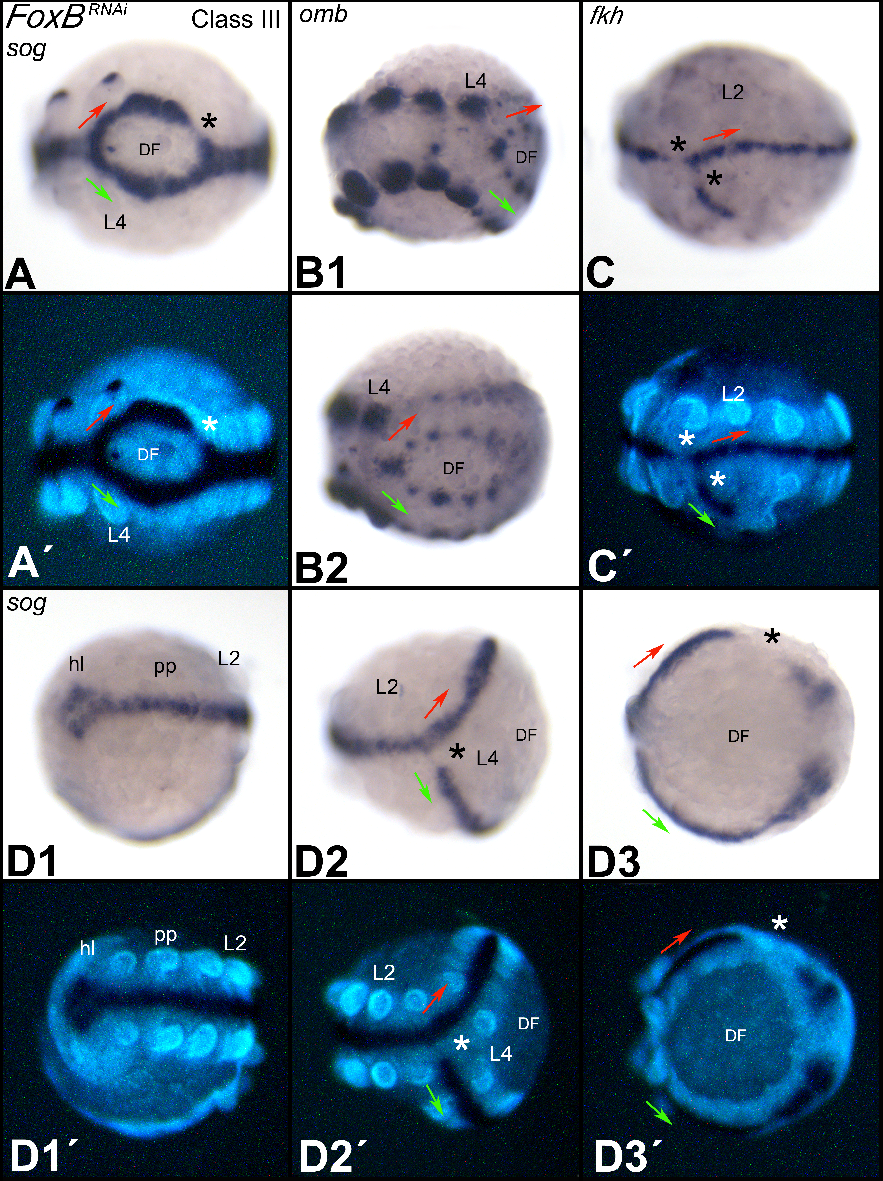

Supplement: Supplementary file 4 — High resolution image (PNG 1536 kb) [file 427_2020_650_Fig7_ESM.png]
